# Population structure of an orchid mycorrhizal fungus with genus-wide specificity

**DOI:** 10.1038/s41598-017-05855-3

**Published:** 2017-07-17

**Authors:** M. P. Ruibal, Y. Triponez, L. M. Smith, R. Peakall, C. C. Linde

**Affiliations:** Division of Ecology and Evolution, Research School of Biology, The Australian National University, Canberra, ACT 2601 Australia

## Abstract

Fundamental life history processes of mycorrhizal fungi with inconspicuous fruiting bodies can be difficult to elucidate. In this study we investigated the species identities and life history of the orchid mycorrhizal *Tulasnella* fungi, which associate with the south eastern Australia orchid genus *Chiloglottis*. *Tulasnella prima* was the primary partner and was found to be associated with all 17 *Chiloglottis* species across a range of >1000 km, and to occur in the two edaphic conditions investigated (soil and sphagnum hammocks). Another *Tulasnella* species (*T*. *sphagneti*) appears to be restricted to moist conditions of alpine sphagnum hammocks. The population genetic structure of the widespread species *T*. *prima*, was investigated at 10 simple sequence repeat (SSR) markers and at four cross-amplified SSR loci for *T*. *sphagneti*. For both taxa, no sharing of multilocus genotypes was found between sites, but clones were found within sites. Evidence for inbreeding within *T*. *prima* was found at 3 of 5 sites. Significant genetic differentiation was found within and between taxa. Significant local positive spatial genetic autocorrelation was detected among non-clonal isolates at the scale of two metres. Overall, the population genetic patterns indicated that in *Tulasnella* mating occurs by inbreeding and dispersal is typically restricted to short-distances.

## Introduction

Many terrestrial orchids interact with compatible mycorrhizal fungi throughout their lifetime. Since orchid seed lack an endosperm and consist only of an embryo and a seed coat, this association is obligate during seed germination with the mycorrhizal fungus supplying the critical nutrients for germination^1, 2^. All orchids continue to rely on fungi for water and nutrients to some extent at adulthood^3^. Investigating the identity and life history of the fungal partners involved in the symbiosis is critical for improving our understanding of fungal biology and the role they play in orchid distribution and abundance^4^.

The identification of fungi, especially mycorrhizal fungi has always been challenging because many species lack distinguishing morphological characters. Consequently species identification as well as the fundamental life history processes that structure mycorrhizal populations such as reproductive strategies, dispersal mechanisms, as well as the spatial distribution of clones, individuals and populations are unknown for the vast majority of fungi^5^. This problem is even more acute for many orchid mycorrhizal fungi (OMF) that mostly belong to the fungal phylum Basidiomycota. Outside of their interaction with orchids, these fungi typically grow saprophytically on dead organic material^6, 7^ where mycelial growth and sexual stages (basidiomata - fruiting bodies) are inconspicuous corticioid structures. To elucidate the life history processes of fungi, knowledge on the occurrence and rate of fruiting (which can differ considerably among fungi)^8^ is important but little is known for OMF.

Despite the wide geographic distribution of OMF spanning eastern and Western Australia^9, 10^, as well as their importance in the recruitment and survival of orchids, nothing is known about their dispersal or mating systems. We predict short dispersal distances for fungi with corticioid fruiting bodies, however mating systems in fungi are complex. Commonly Basidiomycota have two mating type loci (=tetrapolar) or sometimes a single mating type locus only (=bipolar), which control the mating system. The number may vary, but typically fungi in the Basidiomycota have many (up to 1000 s) mating type alleles resulting in many mating types^11^ where fungi with more mating types are expected to have higher levels of outcrossing. Fungi with tetrapolar mating systems are further expected to have more mating types than those with bipolar mating systems (reviewed in ref. 5). Both these mating systems are found in the Agaricomycetes^12^ in which the three common orchid mycorrhizal genera (*Tulasnella*, *Serendipita* and *Ceratobasidium*) are included. It is therefore impossible to predict the mating system or even level of inbreeding/outcrossing of these fungal genera without population genetic investigation.

An unusual pattern of orchid-fungal interactions is found in the fungus *Tulasnella* where just one or two species of fungi is associated with multiple species of Australian terrestrial orchids. In the orchid genera from the tribe Diurideae, subtribe Drakaeinae, the breadth of the association of each orchid genus is restricted to a narrow monophyletic group of lineages of *Tulasnella* fungi, even for orchids collected over several 100–1000 s of kilometres^10, 13, 14^. For example, in *Chiloglottis* (9 spp.) only *Tulasnella prima* Linde & T.W. May and the recently identified *Tulasnella sphagneti* Linde & T.W. May are known as symbionts, while across *Drakaea* and *Caleana* (7 + 5 spp.) *Tulasnella secunda* Linde & T.W. May is the sole known symbiont^10, 15^.

This repeated finding of an Australian orchid genus-wide association each with a narrow phylogenetic mycorrhizal fungal lineage^10, 13, 14^ raises an important question. Is this broad orchid association true, or merely an artefact of the sampling scale and intensity of the present studies? That is, will the pattern dissolve with expanded sampling for fungi across more orchids within the genera concerned with sampling across a larger geographic range and with the inclusion of samples drawn from diverse edaphic conditions?

The orchid genus, *Chiloglottis*, provides a unique opportunity to explore these questions more fully. This predominantly eastern Australian genus consists of 24 described species, with a range spanning from subtropical mid north Queensland, through temperate New South Wales and Victoria, to cooler southern Tasmania. Across this range representatives of the genus inhabit a range of plant communities including rainforest margins, forest, woodlands, coastal heath and subalpine areas that span altitudes from sea level up to 1600 m (Atlas of Living Australia website at http://www.ala.org.au; NSW Flora online at http://plantnet.rbgsyd.nsw.gov.au/). Furthermore, several species can be found growing within the same sites in two contrasting edaphic conditions, soil versus sphagnum hammocks.

In this study we considerably expanded the number of *Chiloglottis* species from which fungal isolates were obtained, with orchid samples drawn from across more than 1000 km, including 17 species, and spanning all the known molecular clades of the genus. In addition, we include an intensive fine-scale analysis (1 m to 30 km) of fungal isolates from three species of *Chiloglottis* that grow in both soil and sphagnum at high altitudes.

Our study is divided into two parts. First, we applied ITS sequencing to determine the phylogenetic diversity of the fungal isolates associated with *Chiloglottis* across our broad sample. Second we conducted a fine-scale population genetic analysis at 10 Simple Sequence Repeat loci (SSR) to investigate the habitat association, mating and life history characteristics of fungal isolates from orchid species associated with soil versus sphagnum. Thus, from the fungal perspective, what are the reproductive and life history characteristics of the fungus that allow it to occupy such a large geographic range and diversity of edaphic conditions? Secondly, from the orchid’s perspective, is edaphic diversification associated with diversification in fungal associations? Our findings confirm the tight association with a narrow phylogenetic diversity of mycorrhizal fungi across *Chiloglottis* orchids, but also confirm a second *Tulasnella* species that is predominantly associated with sphagnum habitats. Our population genetic analyses provide new insights into the fungal biology of the fungal-orchid interaction.

## Results

### *Tulasnella* species diversity and identity

**Figure 1:**
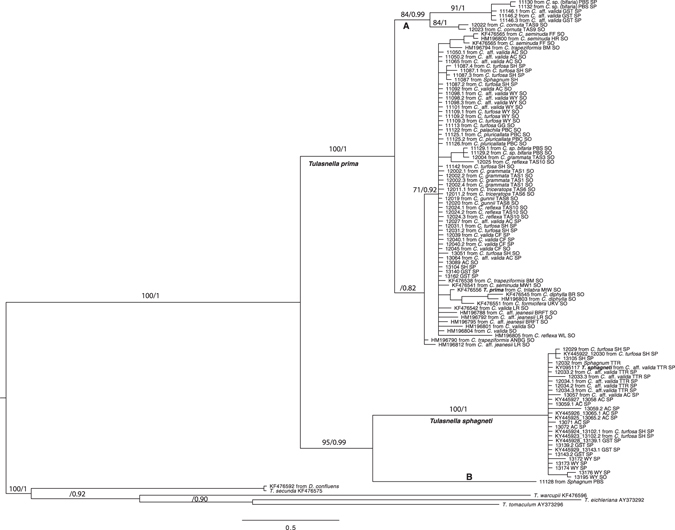
Maximum likelihood of ITS sequences from fungal isolates associated with *Chiloglottis* species. Bayes posterior probability values and bootstrap values are shown. Only posterior probability values >0.70 and bootstrap values >0.80 are shown. For pictorial brevity not all isolates used to compute the posterior probability and bootstrap values were included in the tree. Isolates from the clade representing *Tulasnella prima* were removed from the tree; these isolates were duplicate or representatives isolates taken from the same site and orchid species shown in the tree. Furthermore, *Tulasnella secunda* isolates as well as isolates from *Arthrochilus oreophilus* were removed from the tree for pictorial brevity.

For the ITS phylogenetic analysis, a representative subset of 166 isolates were selected, ensuring coverage of all 17 *Chiloglottis* species and the 28 sampling sites, spanning >1000 km across south eastern Australia. Two sequences obtained from rhizoids of *Sphagnum* spp. were also included (Fig. 1). For visual clarity, a large number of identical sequences were omitted from Fig. 1.

Our analysis found two clades of *Tulasnella* from *Chiloglottis* and a clade represented by a single isolate from a *Sphagnum* rhizoid (clade B) with strong Bayesian Posterior Probabilities (BPP) and bootstrap support (Fig. 1). The largest clade, identified as *T*. *prima* since the type of this species is represented in this clade, represented samples drawn from across all 17 *Chiloglottis* species and from all 28 sites. Furthermore, this *Tulasnella* species was also found in subalpine regions in orchid samples that grew in both soil (n = 37 plants) and sphagnum hammocks (n = 29 plants). All sequences obtained from direct sequencing of *Chiloglottis* peloton rich tissue, were represented in this clade (data not shown).

However, subclade A within *T*. *prima* is also a highly supported clade with high bootstrap (84%) and Bayes Posterior probability (0.99) values. This clade contained isolates taken from *Chiloglottis* orchids growing in sphagnum and soil. To test whether subclade A might be a distinct taxon, we investigated percentage sequence divergences. Clade A recorded a mean K2P sequence divergence of 3.55 ± 0.61 to the main *T*. *prima* clade (clade containing the type) and thus was at the lower limit of sequence divergence for determining whether the sequences represent a different species. A frequency distribution plot of the percentage sequence diversity among isolates (Supplementary Fig. S1) suggests that 5.5 to 6% sequence divergence would be a conservative cut off to delimit species of *Tulasnella* associated with *Chiloglottis*. Similarly, a natural barcode gap across all *Tulasnella* lineages previously investigated were between 4–6% sequence divergence^15^, suggesting that clade A is not a distinct species to *T*. *prima*.

The clade sister to *T*. *prima* was identified as *T*. *sphagneti* since it contained the type of this species. Of the isolates sequenced for ITS, all but 2 of the 44 analysed isolates representing *T*. *sphagneti* were collected from *Chiloglottis* orchids growing in sphagnum hammocks. The two exceptions were collected from *Chiloglottis* growing in soil at the WY site. A sequence obtained from a *Sphagnum* rhizoid also clustered in the *T*. *sphagneti* clade. Another sequence obtained from *Sphagnum* rhizoids at Barrington Tops formed a highly supported sister node (clade B) to *T*. *sphagneti*.

One hundred and thirty ITS haplotypes were observed among the 166 *Tulasnella* isolates from *Chiloglottis*. For *T*. *prima*, 111 haplotypes were observed from 122 isolates. In *T*. *sphagneti*, 26 haplotypes were observed from 44 isolates from *Chiloglottis* and a sequence from *Sphagnum* rhizoids. The mean K2P within taxon sequence divergence (converted to percentage) was 1.35% for *T*. *prima* and 0.26% for *T*. *sphagneti*. The mean sequence divergence between *T*. *prima* and *T*. *sphagneti* was 8.38% across all sites. Mean sequence divergence for clade B against the main clade of *T*. *prima* and *T*. *sphagneti* was 8.11% and 7.92%, respectively. Each of the *Tulasnella* clades from *Chiloglottis* showed between 13.50 to 19.47% sequences divergence to the outgroup *Tulasnella secunda* (Table 1).

**Table 1:** Within host group and between host group Kimura −2P and p-genetic (italicised) distances for *Tulasnella* as calculated from ITS.

	Within taxa	*Tulasnella prima*	*Tulasnella sphagneti*	*Tulasnella* clade B	*Tulasnella secunda*
*Tulasnella prima*	1.35 ± 0.20				
	*1.32* ± *0.19*				
*Tulasnella sphagneti*	0.26 ± 0.06	8.38 ± 1.07			
	*0.26* ± *0.06*	*7.82* ± *0.93*			
*Tulasnella* clade B	—	8.11 ± 1.02	7.92 ± 1.04		
		*7.61* ± *0.90*	*7.38 *± *0.89*		
*Tulasnella secunda*	0.54 ± 0.12	13.50 ± 1.36	15.18 ± 1.49	14.58 ± 1.45	
	*0.53* ± *0.12*	*12.23* ± *1.11*	*13.56 *± *1.19*	*13.13* ± *1.19*	
*Tulasnella warcuppii* + other isolates from *Arthrochilus*	8.46 ± 0.77	17.80 ± 1.49	19.47 ± 1.67	18.41 ± 1.57	15.54 ± 1.35
	*7.68* ± *0.62*	*15.65* ± *1.14*	*16.89* ± *1.25*	*16.16* ± *1.19*	*13.80* ± *1.06*

All 17 *Chiloglottis* orchid species were present among *T*. *prima* isolates (Supplementary Fig. S2) suggesting there is no fungal-*Chiloglottis* host specificity. *T*. *sphagneti* was restricted to two orchid host species; *C*. *turfosa* and *C*. aff. *valida*, but possibly also *C*. *valida*, and only when these hosts were growing in sphagnum hammocks (one exception). A single sample was from *Sphagnum* rhizoids. Clade B was represented by a single isolate obtained from sphagnum rhizoids, although not from *C*. sp. (bifaria) orchids which were growing nearby (separated by 2–3 meters only).

### Between species population genetic structure of *Tulasnella* at SSR loci

**Figure 2:**
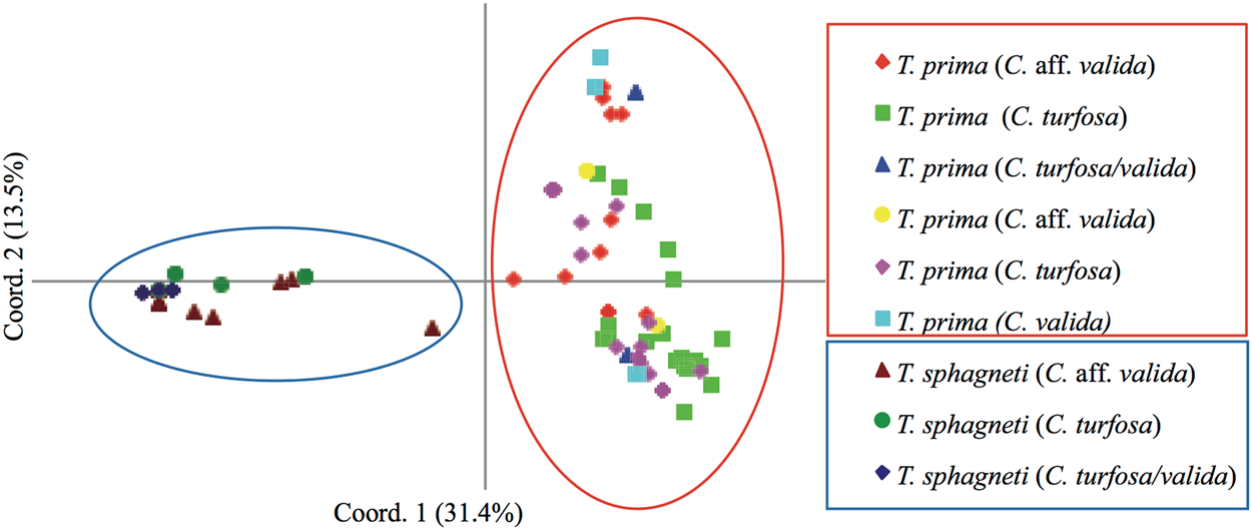
PCoA plot for *Tulasnella prima* and *Tulasnella sphagneti* at four SSR loci.

In the SSR analyses, *T*. *prima* (66 MLGs) amplified successfully with all 10 SSR loci while in *T*. *sphagneti* (33 MLGs) only four of the 10 loci amplified successfully. The hierarchical AMOVA analysis of the SSR data revealed extensive differentiation between *T*. *prima* and *T*. *sphagneti* (Table 2), with 44% of the total genetic variance partitioned among the species across the 10 loci (*F*_*st*_ = 0.569, six loci set to fixed differences for *T*. *sphagneti*). As expected, across the four shared loci, the degree of genetic differentiation between the species was marginally lower (33%) (Table 3). Little allelic sharing occurred among *T*. *prima* and *T*. *sphagneti* at the four amplifiable SSR loci in common (Supplementary Fig. S3). These two species formed separate clusters in the PCoA with no clustering with respect to orchid species within either *Tulasnella* species (Fig. 2).

**Table 2:** Hierarchical AMOVA across 10 SSR loci with six of 10 loci set to a fixed difference for *Tulasnella sphagneti*.

*F*_*st*_	df	SS	MS	Est. var.	%	*P* value
Among *T*. *prima* and *T*. *sphagneti*	1	261.80	261.80	2.85	54%	0.001
Among sites	8	67.48	8.44	0.30	6%	0.001
Among isolates	90	246.51	2.74	0.59	11%	
Within isolates	100	157.00	1.57	1.57	30%	
Total	199	732.80		5.31	100%

**Table 3:** Hierarchical AMOVA for four loci amplified for *Tulasnella prima* and *Tulasnella sphagneti*.

*F*_*st*_	df	SS	MS	Est. var.	%	*P* value
Among *T*. *prima* and *T*. *sphagneti*	1	65.05	65.05	0.67	33%	0.001
Among sites	8	39.11	4.89	0.19	9%	0.001
Among isolates	90	115.60	1.28	0.10	5%	
Within isolates	100	108.50	1.09	1.09	53%	
Total	199	328.26		2.05	100%

### Within population genetic structure of *Tulasnella* at SSR loci

Within the two *Tulasnella* species, significant genetic differentiation was detected among populations (6% across the 10 loci, 9% across four loci). A hierarchical multilocus analyses of molecular variance for *T*. *prima* from Kosciuszko National Park revealed that most of the variation is found within individuals (59%), with a large portion of the remaining variation explained by variation among individuals (29%) and some by among population differences (13%). However, the reduced number of amplifiable loci for *T*. *sphagneti* meant that we could not include this species in all population genetic analyses.

### Determination of unique identity of multilocus genotypes

**Figure 3:**
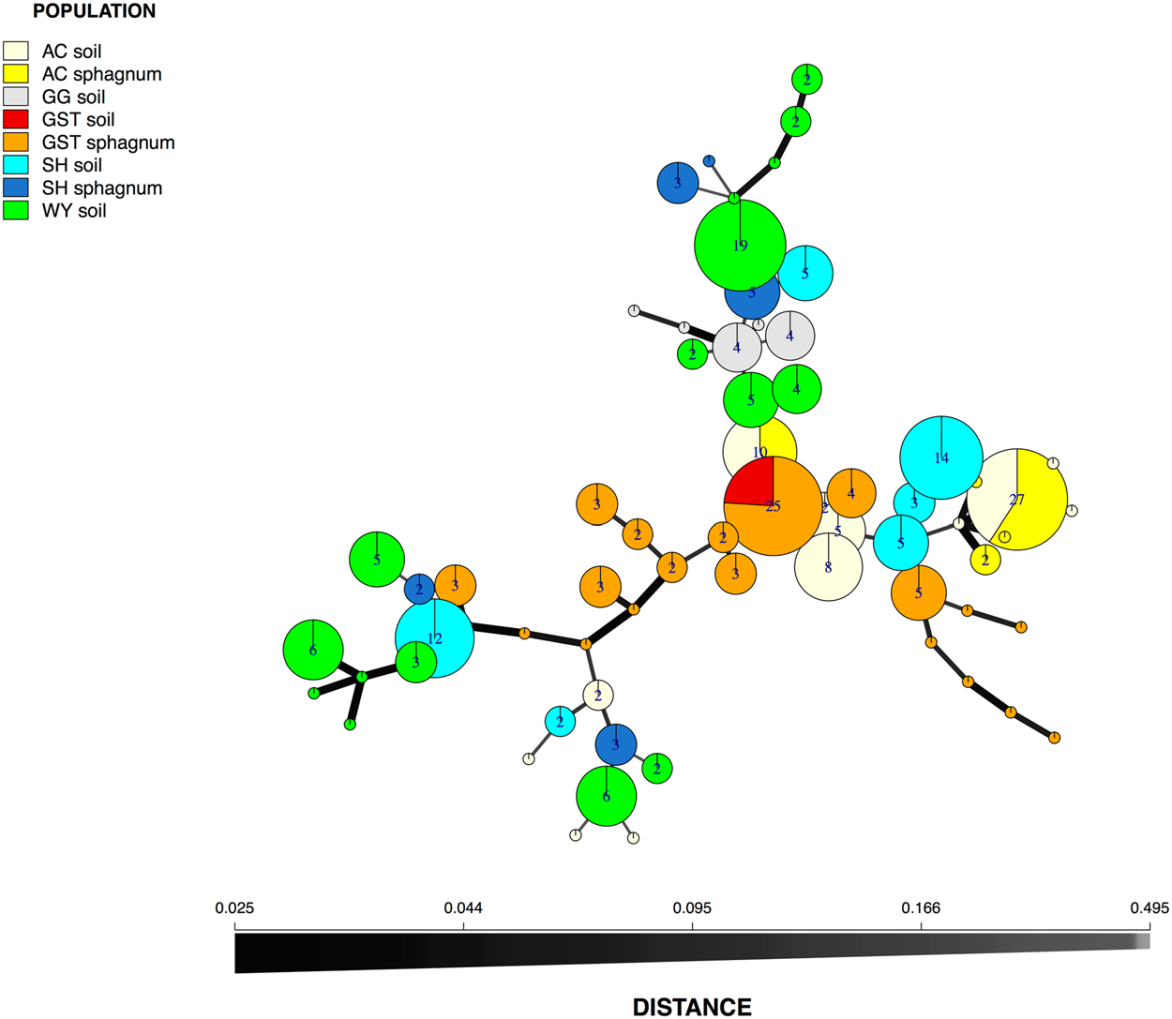
Minimum spanning network showing the relationship between individual SSR multilocus genotypes (MLGs) for *Tulasnella prima* observed in isolates collected in Kosciuszko National Park, NSW from *Chiloglottis* plants growing in soil or sphagnum hammocks. Each node represents a different MLG. Node sizes and colours correspond to the number of individuals and membership, respectively. The number of individuals per MLG greater than one is provided. Edge thickness and colour are proportional to absolute genetic distances.

For *T*. *prima* from the more intensively sampled area within Kosciuszko National Park, NSW, we genotyped 253 isolates from 99 plants sampled across 5 sites (AC, GST, WY, SH, GG), with an average of two isolates per plant (range 1 to 10 isolates per plant). The conservative *PI*_*sibs*_ was estimated to be <0.001 across the 10 SSR loci (Supplementary Fig. S4), indicating strong power to distinguish among different genetic individuals. From our sample of 253 isolates, we detected 67 multilocus genotypes (MLGs), with just seven of the 10 loci required to resolve all 67 MLGs. Therefore, duplicate MLGs were presumed to be clones. Such clones were only found within sites and never shared between sites (Fig. 3), with the most frequently observed clone (27 isolates from 16 plants) at site AC covering an area of over 150 m^2^. Three MLGs could be isolated from plants growing in either soil or sphagnum at all three sites where *T*. *prima* was isolated from plants growing in both soil and sphagnum. We found 13 of the 99 plants had more than one fungal MLG, in all 13 cases the same species of *Tulasnella* was involved.

Within *T*. *sphagneti*, we genotyped 33 isolates across five sites (AC, GST, WY, SH, TTR). Probability of unique identity for the 4 amplifiable loci among unrelated (*PI*) genotypes was *P* > 0.045 and for related (*PI*_*sibs*_) genotypes *P* > 0.227; suggesting that to identify MLGs, five or more polymorphic loci would be required among unrelated isolates and a greater number for related isolates.

### Mating system

Within *T*. *prima* from the Kosciuszko National Park, two sites (GG and SH) were in HWE, while three sites showed heterozygote deficiencies. Across all loci and individual sites, there were 22 (of 50) deficits of heterozygotes with significant departures (*P* < 0.05). After Bonferroni correction, 12 (of 50) loci remained as having a significant heterozygote deficit (Table 4 and Supplementary Table S2). Only two loci from GST showed excess heterozygosity with significantly (*P* < 0.001) negative *F* values. Positive and significant (*P* < 0.001) mean *F* values were recorded for three sites (AC, GST, WY) showing a deficit of heterozygous individuals compared with expectations under random mating (Table 4), suggesting inbreeding occurs within these sites. When tests of HWE were assessed for the *T*. *prima* isolates from Kosciuszko as a single population, we found that all loci significantly (*P* < 0.001) departed from HWE, as a result of a deficit of heterozygotes (data not shown).

**Table 4:** Measures of SSR diversity for *Tulasnella prima* sampled at sites in Kosciuszko National Park: Number of isolates (N), number of alleles (Na), observed heterozygosity (*H*_*o*_), expected heterozygosity (*H*_*e*_), unbiased expected heterozygosity (*uH*_*e*_), fixation index (*F*) and significance for Hardy-Weinberg equilibrium before and after Bonferroni correction.

Site	Locus	N	Na	*H*_*o*_	*H*_*e*_	*uH*_*e*_	*F*	HWE	HWE (Bonferroni)
**AC**	Tul 2	15	4	0.467	0.542	0.561	0.139	*	ns
	Tul 11	15	7	0.800	0.711	0.736	−0.125	*	ns
	Tul 12	15	3	0.800	0.560	0.579	−0.429	ns	ns
	Tul13	15	3	0.600	0.647	0.669	0.072	**	ns
	Tul 16	15	6	0.267	0.711	0.736	0.625	***	***
	Tul 17	15	2	0.467	0.420	0.434	−0.111	ns	ns
	Tul 23	14	5	0.071	0.717	0.743	0.900	***	***
	Tul 24	15	1						
	Tul 65	15	1						
	Tul TGC6	15	4	0.133	0.293	0.303	0.545	*	ns
*Mean*				*0*.*360*	*0*.*460*		*0*.*202*		
**GG**	Tul 2	5	2	0.200	0.180	0.200	−0.111	ns	ns
	Tul 11	5	4	0.400	0.580	0.644	0.310	ns	ns
	Tul 12	5	2	0.800	0.480	0.533	−0.667	ns	ns
	Tul13	5	2	1.000	0.500	0.556	−1.000	*	ns
	Tul 16	4	2	1.000	0.500	0.571	−1.000	*	ns
	Tul 17	5	2	0.200	0.180	0.200	−0.111	ns	ns
	Tul 23	4	4	0.500	0.656	0.750	0.238	ns	ns
	Tul 24	5	1						
	Tul 65	5	2	0.200	0.180	0.200	−0.111	ns	ns
	Tul TGC6	5	3	0.200	0.460	0.511	0.565	ns	ns
*Mean*				*0*.*450*	*0*.*372*		−*0*.*210*		
**GST**	Tul 2	20	4	0.650	0.681	0.699	0.046	***	***
	Tul 11	20	4	1.000	0.734	0.753	−0.363	***	***
	Tul 12	20	4	0.850	0.746	0.765	−0.139	***	***
	Tul13	18	7	0.667	0.779	0.802	0.145	***	***
	Tul 16	16	9	0.438	0.863	0.891	0.493	**	ns
	Tul 17	20	4	0.100	0.516	0.529	0.806	**	ns
	Tul 23	13	5	0.154	0.710	0.738	0.783	***	***
	Tul 24	20	2	0.050	0.469	0.481	0.893	***	***
	Tul 65	20	2	0.000	0.455	0.467	1.000	***	***
	Tul TGC6	20	6	0.400	0.630	0.646	0.365	***	***
*Mean*				*0*.*431*	*0*.*658*		*0*.*403*		
**SH**	Tul 2	10	5	0.800	0.735	0.774	−0.088	ns	ns
	Tul 11	10	6	0.800	0.775	0.816	−0.032	ns	ns
	Tul 12	10	7	0.900	0.790	0.832	−0.139	ns	ns
	Tul13	10	3	0.500	0.395	0.416	−0.266	ns	ns
	Tul 16	7	6	0.571	0.755	0.813	0.243	ns	ns
	Tul 17	10	3	0.300	0.405	0.426	0.259	**	ns
	Tul 23	10	3	0.200	0.545	0.574	0.633	ns	ns
	Tul 24	10	1						
	Tul 65	10	2	0.100	0.095	0.100	−0.053	ns	ns
	Tul TGC6	10	3	0.500	0.515	0.542	0.029	ns	ns
*Mean*				*0*.*467*	*0*.*501*		*0*.*065*		
**WY**	Tul 2	17	3	0.353	0.519	0.535	0.320	***	***
	Tul 11	15	4	0.333	0.473	0.490	0.296	*	ns
	Tul 12	17	4	0.471	0.678	0.699	0.306	**	ns
	Tul13	17	4	0.647	0.623	0.642	−0.039	*	ns
	Tul 16	11	7	0.727	0.831	0.870	0.124	**	ns
	Tul 17	17	3	0.294	0.413	0.426	0.289	***	***
	Tul 23	15	4	0.067	0.624	0.646	0.893	***	***
	Tul 24	17	2	0.000	0.111	0.114	1.000	***	***
	Tul 65	16	2	0.125	0.117	0.121	−0.067	ns	ns
	Tul TGC6	16	4	0.500	0.557	0.575	0.102	*	ns
*Mean*				*0*.*352*	*0*.*495*		*0*.*322*		

Key: ns = not significant, *P < 0.05, **P < 0.01, ***P < 0.001.

In *T*. *sphagneti*, two of the four loci were monomorphic for each site (Tul 2 and Tul 16), and only sites GST and SH showed HWE at the two other amplifiable loci (Tables 5 and S3). After Bonferroni correction, only Tul 12 showed a significant (*P* < 0.01) heterozygote deficit (Table 5). With all isolates grouped as a single population only one locus (Tul 11) of the three polymorphic loci was in HWE with Tul 12 and Tul 16 significantly (*P* < 0.001) departing from HWE (data not shown).

**Table 5:** Measures of SSR diversity for *Tulasnella sphagneti* sampled at Kosciuszko National Park: Number of isolates (N), number of alleles (*Na*), observed heterozygosity (*H*_*o*_), expected heterozygosity (*H*_*e*_), unbiased expected heterozygosity (*uH*_*e*_), fixation index (*F*) and significance for Hardy-Weinberg equilibrium before and after Bonferroni correction.

Pop	Locus	N	Na	*H*_*o*_	*H*_*e*_	*uH*_*e*_	*F*	HWE	HWE (Bonferroni)
**AC**	Tul 2	13	1	0.000	0.000	0.000			
	Tul 11	14	4	1.000	0.564	0.585	−0.774	*	ns
	Tul 12	7	2	1.000	0.500	0.538	−1.000	**	**
	Tul 16	14	1	0.000	0.000	0.000			
*Mean*				*0*.*500*	*0*.*266*		−*0*.*443*		
**GST**	Tul 2	3	1	0.000	0.000	0.000			
	Tul 11	3	2	1.000	0.500	0.600	−1.000	ns	ns
	Tul 12	3	2	1.000	0.500	0.600	−1.000	ns	ns
	Tul 16	3	1	0.000	0.000	0.000			
*Mean*				*0*.*500*	*0*.*250*		−*0*.*500*		
**SH**	Tul 2	5	1	0.000	0.000	0.000			
	Tul 11	5	2	0.600	0.420	0.467	−0.429	ns	ns
	Tul 12	5	3	1.000	0.620	0.689	−0.613	ns	ns
	Tul 16	5	2	0.200	0.180	0.200	−0.111	ns	ns
*Mean*				*0*.*450*	*0*.*305*		−*0*.*288*		
**WY**	Tul 2	9	1	0.000	0.000	0.000			
	Tul 11	9	3	0.778	0.537	0.569	−0.448	ns	ns
	Tul 12	9	2	1.000	0.500	0.529	−1.000	**	**
	Tul 16	9	1	0.000	0.000	0.000			
*Mean*				*0*.*444*	*0*.*259*		−*0*.*362*		

Key: ns = not significant, *P < 0.05, **P < 0.01, ***P < 0.001.

### Patterns of genetic structure associated with fungal species, geographic origin, edaphic conditions and host identity

Pairwise site *F*_*st*_ values ranged from 0.051–0.216 for *T*. *prima* and 0.060 to 0.545 for *T*. *sphagneti*. All within taxa pairwise site comparisons were significantly different, except those between sites SH and WY (Table 6); these two sites were approximately 7.3 km apart which is at the low end of the geographic distances between sites (5.8 to 26 km). Pairwise *F*_*st*_ values within *T*. *sphagneti* for site GST were particularly high, which was due to the presence of a private allele at locus Tul 12 that was shared by all GST isolates.

**Table 6:** Pairwise *F*_*st*_ results for within (shaded areas) and among *Tulasnella* taxa for Kosciuszko National Park sites.

		*Tulasnella prima*					*Tulasnella sphagneti*			
		AC	GG	GST	SH	WY	AC	GST	SH	WY
*Tulasnella prima*	AC		***	***	***	***	***	***	***	***
	GG	0.216		***	***	***	***	***	***	***
	GST	0.159	0.110		***	***	***	***	***	***
	SH	0.127	0.165	0.104		***	***	***	***	***
	WY	0.102	0.073	0.098	0.051		***	***	***	***
*Tulasnella sphagneti*	AC	0.478	0.591	0.411	0.433	0.468		***	*	***
	GST	0.416	0.577	0.354	0.354	0.402	0.502		**	***
	SH	0.423	0.562	0.362	0.365	0.414	0.097	0.513		**0.164**
	WY	0.476	0.637	0.414	0.440	0.468	0.166	0.545	0.060	

All 10 microsatellite loci were included for *Tulasnella prima* pairwise site comparisons, and four microsatellite loci for among taxa comparisons, and within *Tulasnella sphagneti* pairwise site comparisons. Bottom triangle shows *F*_*st*_ values and top triangle shows probability values for *F*_*st*_, non-significant probabilities are highlighted in bold.

***P values from 0.001, **≤0.025, *≤0.04.

When similar sampling scales were compared for *T*. *prima* and *T*. *sphagneti* in the Kosciuszko NP region (sites up to 16 km apart), a strong positive and significant relationship was detected between geographic and genetic distance for both species (*rxy* = 0.225, *P* ≥ 0.001 and *rxy* = 0.577, *P* ≥ 0.001 respectively). Interestingly, across *T*. *prima* sampled from the Kosciuszko NP region (sites up to 26 km apart), no individual level isolation by distance was detected (*rxy* = 0.113, *P* ≥ 0.065), nor for the Australia wide dataset.

At the three sites where *T*. *prima* isolates were sampled from both soil and sphagnum, no significant genetic differentiation was found between the two habitat groups of isolates at two sites (SH- *F*_*st*_ 0.068, *P* ≥ 0.050; AC- *F*_*st*_ 0.022, *P* ≥ 0.188), but significant genetic differentiation was found at site GST (*F*_*st*_ 0.133, *P* ≥ 0.017), which is likely an artefact of the differences in sample size (N = 4 from soil, N = 17 from *Sphagnum*). Within *T*. *prima*, the overlay of geographic origin onto the minimum spanning SSR networks revealed that genotype sharing was geographically restricted (Supplementary Fig. S5). However, there was no clustering associated with orchid species identity (Supplementary Fig. S2). Similarly, no clustering of genotypes for sphagnum versus soil was found within or among sites (Fig. 3).

Isolates which were ITS sequenced and identified as *T*. *sphagneti* clustered together in a PCoA with the 15 *Tulasnella* isolates which were SSR typed only (Fig. 2), suggesting they all belong to *T*. *sphagneti*. All but two of the 44 isolates (or 24 of the 26 *Chiloglottis* plants) representing *T*. *sphagneti* were collected from *Chiloglottis* orchids growing in sphagnum hammocks. The two isolates collected from plants growing in soil came from a single site (WY), however an intensive bushfire five years prior to the collection date may have caused the recession of sphagnum from the area, suggesting a sphagnum habitat specificity for *T*. *sphagneti*.

### Fine-scale within site genetic structure

**Figure 4:**
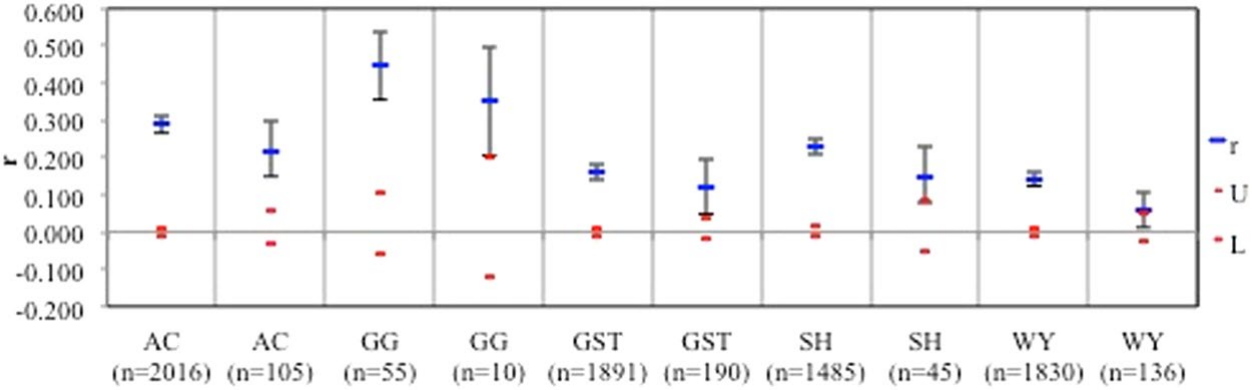
The figure shows the autocorrelation (*r*) values among five sites located at Kosciuszko National Park for datasets with clones included and clone corrected, respectively. The 95% confidence intervals about the *r* value were determined via bootstrapping and upper and lower bounds for the 95% confidence intervals about the null hypothesis of no genetic structure (*r* = 0) determined by random permutation.

**Figure 5:**
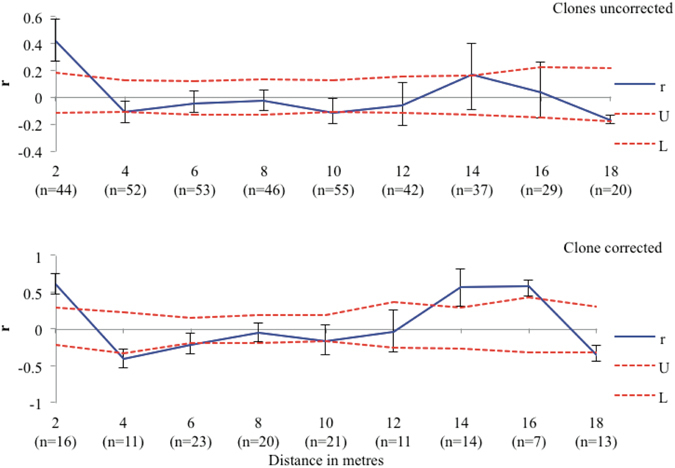
Spatial autocorrelation for site GST located at Kosciuszko National Park for datasets with clones included and clone corrected. The 95% confidence intervals about the *r* value were determined via bootstrapping and upper and lower bounds for the 95% confidence intervals about the null hypothesis of no spatial structure (*r* = 0) determined by random permutation.

At each of the five sites in Kosciuszko National Park, spatial autocorrelation analyses for *T*. *prima* isolates showed significant and positive autocorrelation values for the clone uncorrected (range *r* = 0.142–0.452) or corrected (range *r* = 0.06–0.353) datasets (Fig. 4). At all five sites the autocorrelation values were greater with clones than without. Fine scale genetic structure at the within-site level with a distance class of two metres showed that *r* is positive and significant at the shortest distance class of two metres and then generally declines for both the clone uncorrected and corrected datasets at site GST (Fig. 5).

## Discussion

Building on our previous work, this study represents the largest and most comprehensive study of orchid-fungal associations across any orchid genus. In total, mycorrhizal fungi from 17 *Chiloglottis* species representing 28 sampling sites, spanning all four subclades and >1000 km across southeastern Australia, were investigated. Our study also captured a representative number of *Chiloglottis* orchid species growing in the same area but in different habitats (soil vs sphagnum hammocks), representing intensive sampling at small spatial scales (within site <1 m–15 m; within regions 5 to 10’s of km), as well as regional comparisons (between regions >200 km to over 1000 km).

### Mycorrhizal diversity associated with the genus *Chiloglottis*

Consistent with our previous studies on a limited number of *Chiloglottis* species^10, 14^, all 17 *Chiloglottis* orchid species investigated associated with a dominant *Tulasnella* species, *T*. *prima*. A second and recently discovered *Tulasnella* species, *T*. *sphagneti* ^15^, was found associated with orchids that grow in subalpine sphagnum hammocks. ITS sequence divergence between *T*. *prima* and *T*. *sphagneti* (8.38% K2P) fell well above the widely accepted 3% sequence divergence cut-off value for species delimitation^16^, and the 3–5% divergence proposed for delimiting *Tulasnella* species^17–19^. Direct sequencing of peloton rich tissues from *Chiloglottis* plants in this and a previous study^14^, did not reveal additional orchid mycorrhizal fungal lineages to the cultured isolates, indicating that unculturable fungi are likely to be negligible in this study system. A *Ceratobasidium* isolate was isolated once and found twice with direct sequencing, however, germination tests with this isolate (data not shown) failed to germinate *Chiloglottis* seed and hence it is likely a contaminant.

This orchid mycorrhizal association of limited fungal diversity with an entire plant genus, is in stark contrast to the generalist arbuscular mycorrhizae and to a somewhat lesser degree ectomycorrhizae, which all have broad host ranges^20^. *Tulasnella prima* has not been found to associate with any other orchid host and thus appears to be restricted to one genus. In general, orchids tend to associate with phylogenetically related groups of fungi. For example, typically an orchid genus associates with several operational taxonomic units (OTUs) of either *Tulasnella*, *Serendipita* or *Ceratobasidium* ^21–26^. Although such associations are interpreted as “specific”, the level of specificity we have discovered in the *Tulasnella-Chiloglottis* system appears to be unprecedented. In studies of European terrestrial orchids, 15 OTUs from the Tulasnellaceae were associated with four species of *Anacamptis*. Thirteen of these 15 OTUs were associated with seven *Ophrys* and two *Orchis* species and nine OTUs with three *Serapias* species^27^. Furthermore, within sites, up to 15 OTUs were co-occurring and 85% of orchid plants associated with more than three different OTUs^27^. A corresponding result was found along a single 1000 m transect in Italy where 16 Tulasnellaceae OTUs were recovered for 20 species of orchids from the genera *Anacamptis*, *Neotinea*, *Ophrys*, *Orchis*, and *Serapias* ^28^. Similarly, low specificity in another study showed up to seven *Tulasnella* OTUs may associate with *Orchis* species within single populations^23^. The same pattern is found in Andean tropical rainforests where up to 6 *Tulasnella* OTUs may associate with *Stelis* orchid species and *Pleurothallis lilijae* ^26, 29^. Consistent among these and our studies, is the finding that multiple species of an orchid genus can share the same fungal OTU.

Our findings raise the question of why only a small diversity of *Tulasnella* fungi associate with an Australian orchid genus spanning a vast geographic range, and across a diversity of habitats. The only other finding of such narrow diversity is found in Australian orchids belonging to the same subtribe (Drakaeinae) as *Chiloglottis*, ie *Drakaea* and *Caleana* (1 OTU in 7 *Drakaea* and 5 *Caleana* spp.)^10, 13^. One exception in this orchid subtribe is found in *Arthrochilus oreophilus* where 3 OTUs occur in a narrow sample of this subtropical species^10^. In contrast to the Drakaeinae, five species of *Diuris* (Tribe Diurideae, subtribe Diuridinae) in Australia associate with several OTUs^30^.

Currently we have little understanding of why some orchid species interact with a single fungal OTU and other species associate with multiple OTUs. Evolutionary and ecological constraints may account for the patterns of association. Martos *et al*.^31^ suggest that where there is asymmetry in associations, phylogenetically constrained heritable traits in the plants may control the suitability of the fungal partner. Furthermore, if related orchids retain much of their ancestral ecological niche, they are likely exposed to the same suite of mycorrhizal fungi^31^. Some evidence supporting this hypothesis is found in our study, given *T*. *sphagneti* was only present in three orchid species that occupy the different edaphic conditions of sphagnum hammocks, whereas all the other species occur in soil and associate with the common and widespread *T*. *prima*.

The wide distribution of a common species such as *T*. *prima* also may reflect the stable geology of Australia and its associated nutrient poor soils^32^. By contrast, Europe has had multiple glaciation cycles^33^ and the soils are comparatively nutrient rich. There is evidence that edaphic conditions and competitiveness influence fungal diversity (see reviews)^34, 35^; hence in poor soils some fungal species may outcompete less abundant species, whereas in nutrient rich soils sufficient nutrients may sustain a greater diversity of fungi.

### Habitat-driven mycorrhizal associations

Whereas *T*. *prima* was found in *Chiloglottis* orchids growing in soil (in *Eucalyptus* woodlands and forests, subalpine areas) as well as those growing in sphagnum hammocks of subalpine areas, *T*. *sphagneti* was strongly associated with *Chiloglottis* orchids only when they were growing in subalpine sphagnum hammocks. The strong habitat preference of *T*. *sphagneti* suggests this fungus is restricted to cool wet environments typical of sphagnum hammocks. Until very recently, the role of habitat as a driver for orchid mycorrhizal differentiation has not been investigated. The first orchid-mycorrhizal study to show habitat preference was on *Neottia ovata*, which showed that mycorrhizal communities differ substantially between grassland and forest populations of the orchid^36^. Similarly, habitat was shown to drive different mycorrhizal communities in *Epipactis* orchids growing in dune slacks vs forests^37^ and in *Dactylorhiza* orchids^4^ in Europe. Our findings reinforce the growing evidence for habitat-driven mycorrhizal associations in orchids and suggest future studies may find this to be a common trend.

Hosts of orchid-mycorrhizae other than the orchid itself are rarely identified. Here we discovered that the three *Tulasnella* species (*T*. *prima*, *T*. *sphagneti* and *Tulasnella* clade B) isolated from orchids also occur in *Sphagnum* rhizoids (Fig. 1). Aneuraceae thalloid liverworts are known to form associations with *Tulasnella* in a relationship that may be analogous to mycorrhizas of vascular plants^38, 39^, however until now associations with *Sphagnum* have not been investigated. *Sphagnum* and Bryophytes in general may harbour a vast diversity of *Tulasnella* that were previously undetected. Interestingly, the three *Tulasnella* lineages are closely related which may suggest a phylogenetic constraint on host associations and habitat.

### Population genetic structure of *T*. *prima*

To date, few studies on mycorrhizal fungi and none on orchid-mycorrhizal fungi employed fine-scale spatial analyses of genetic structure to infer the mating system or dispersal. Examining genetic structure at the scale of metres to 100’s of metres allows us to infer the biological processes that lead to positive or negative patterns of genetic distribution.

For *Tulasnella*, we analysed vegetative material that is dikaryotic (two SSR alleles per locus in an individual) where the two parental haploid nuclei co-exist. Unfortunately only four SSR loci consistently amplified for *T*. *sphagneti*, preventing meaningful conclusions regarding its population genetic structure. For *T*. *prima*, the SSR markers revealed highly localised genetic structure within sites, along with the trend for heterozygote deficiencies with positive *F* values reflecting localised inbreeding. Furthermore, three of the tested five populations showed significant deviations of genotype frequencies from Hardy-Weinberg expectations and high positive *F* values, especially for population GST, consistent with prevalent inbreeding within populations. Only two populations were in HWE, suggesting random mating. Inbreeding is inferred because heterozygote deficiencies were not restricted to certain loci, but was population specific^40^. However, non-random sampling where homozygotes may have a higher probability of being associated with orchid hosts, or selection against migrants^41, 42^, as a cause for heterozygote deficiencies^40^ is probable, but highly speculative in the absence of supporting evidence for such rare phenomena in fungi.

Inbreeding (positive assortative mating) arises when there is mating of related individuals within a population, resulting in individuals being more related than expected under random mating. Although the complex mating systems in basidiomycetes have evolved to promote outcrossing, selfing may occur within a dikaryon that consists of nuclei with compatible mating type alleles. Thus in such cases, in a tetrapolar mating system, 25% of all basidiospores from a single mating event (cluster of basidia) may fuse with a parental haploid individual/genotype (reviewed in ref. 5). Generally, saprotrophic mushrooms have randomly mating populations, but inbreeding is shown increasingly in various mushrooms ranging from the ectomycorrhizal fungus *Laccaria amethystina* ^43^, the saprophytic mushrooms *Trogia venenata* ^44^ and the bird’s nest fungus *Cyathus stercoreus* ^45^. Although further investigation is required to determine whether *Tulasnella* has a bipolar or tetrapolar mating system, the high levels of inbreeding detected in this study suggest a few mating types only in *T*. *prima*.

Significant genetic differentiation (*F*_*st*_ = 0.051–0.216) between sites and high spatial autocorrelation found over very short distances (3 m), supports short distance dispersal of basidiospores. Most basidiospores, even among some wind-dispersed fungi, are thought to fall close to the source, which would result in the clustering of genetically similar isolates. Indeed in wind dispersed mushroom species significant positive genetic structure at local scales of 1 to 20 metres^46, 47^ and up to 400 m^48^ has been reported. Nonetheless, the signature of spatial clustering among some sites within Kosciuszko suggests that some long distance dispersal of basidiospores does occur, albeit infrequently. It is important to note that estimates of genetic structure produced through *F*-statistics reflect evolutionary signals of interconnection through gene flow^49^, whereas spatial autocorrelation provide contemporary genetic signatures. Consequently our results showing restricted short distance dispersal and infrequent long distance dispersal are consistent with studies that have found that most basidiospores tend to fall close to the parent and few are carried long distances via wind dispersal.

Although it is problematic to compare *F*_*st*_ values across studies without standardising *F*_*st*_^50^, we have found that the level of genetic differentiation between *Tulasnella* populations in our study were higher at comparable geographic distances than observed in other Basidiomycota fungi population genetic analyses (see review by ref. 5). Basidiomata (fruiting structures) of *Tulasnella* associated with *Chiloglottis* have not been observed and it possible that basidiomata of *Tulasnella* are rarely formed. If this is the case, rare fruiting could severely limit basidiospore production and associated gene flow. Indeed, our population genetic findings suggest that clonal expansion, via vegetative growth or fragmentation of mycelium aided by animal dispersal, seems to be the predominate means of dispersal in the study species.

In conclusion, our new findings are consistent with previous studies confirming that across the genus of *Chiloglottis*, all orchid species associate with the common and widespread fungus *T*. *prima*. However, the species of *Tulasnella* involved in this orchid-interaction can be influenced by the edaphic conditions in which the orchid grows. Under wetter conditions associated with sphagnum hammocks, a second *Tulasnella* fungus (*T*. *sphagneti*) may co-occur with the generalist *T*. *prima*. Our results also provide the first insights into the mating system and likely extent of dispersal within *Tulasnella*. Despite the wide geographic distribution of *T*. *prima* the population genetic results strongly indicate that this species predominantly disperses over short distances and mostly via vegetative (asexual) fragments or predominantly inbred basidiospores.

## Methods

### Orchid sample collection for mycorrhizal isolation, *Tulasnella* species diversity and identity

For this first component of the study, *Tulasnella* mycorrhizal associations were investigated in 17 species of *Chiloglottis*, representing the three main genetic clades of the genus^51^: Formicifera clade (C. *trapeziformis*, *C*. *formicifera*); Reflexa clade (*C*. *reflexa*, *C*. *trilabra*, *C*. *seminuda*, *C*. *diphylla*); Valida clade (*C*. *turfosa*, *C*. *valida*, *C*. *cornuta*, *C*. *grammata*, *C*. *jeanesii*, *C*. *pluricallata*, *C*. *triceratops*, *C*. *gunnii*). Two undescribed species were included from the Valida clade, for which there is strong pollinator, chemical and genetic evidence that they are distinct, but morphologically cryptic species: *C*. aff. *valida* and *C*. sp. (bifaria)^51–53^. *Chiloglottis palachila* was also included as the sole representative of a fourth subclade^51, 52^.

Orchid plant samples were collected from sites spanning >1000 km across south-eastern Australia. In combination with the earlier sampling efforts of Roche *et al*.^14^ and Linde *et al*.^10^, our final collection covered a total of 28 sites in New South Wales (NSW), Australian Capital Territory (ACT) and Tasmania (TAS), Australia (Supplementary Table S1).

### Orchid sample collection for population structure of *Tulasnella* at SSR loci

Most of the *Chiloglottis* species within the Valida clade grow in montane to subalpine regions where they can be observed growing both in moist to wet peaty soils with sphagnum, as well as in soil away from sphagnum dominated habitats. Thus this clade provides a unique opportunity to compare fungal interactions within orchid species that grow under contrasting edaphic conditions, sometimes separated by a few metres only. We therefore sampled three species from this group: *C*. *valida*, *C*. aff. *valida* and *C*. *turfosa*. These species were sampled from both soil and sphagnum hammocks across each of 5 sites (AC, GST, WY, SH, GG) within the Kosciuszko National Park, NSW.

As for the orchid genus generally, flowering in these three species is sparse, with populations typically consisting of multiple colonies each with 100’s to 1000’s of plants, but few if any with flowers. The three species cannot be reliably distinguished by leaf morphology. Furthermore, *C*. *valida* and *C*. aff. *valida* flowers are morphologically indistinguishable^53^, but distinct from *C*. *turfosa*. Therefore, it was only possible to provisionally identify our samples to species based on floral morphology (when available) and on prior chemical and genetic analysis of tagged colonies from the earlier study of Peakall and Whitehead^53^.

Depending on the shape of the orchid distribution at a particular site, we used either transects or grids to sample the plants. A single orchid plant located approximately every 1 to 2 metres apart was collected resulting in 30–51 plants collected per Kosciuszko population. Individual plants were mapped for one population (GST) to allow fine-scale analyses of genetic relatedness among fungal isolates.

#### Fungal isolation

Isolations were conducted within 7 days of the field collection of the plant tissue using a modified version of the protocol of Roche *et al*.^14^. We used two types of isolation media to grow mycorrhizal isolates: Fungal Isolation Media (FIM)^54^ and 3MN + A-Z, which is a Melin-Norkrans medium (low CN MMN)^55^ modified with 15 g.l^−1^ agar as well as human vitamin and mineral supplements (Centrum “Balanced Formula”, Wyeth Consumer Healthcare, Baulkham Hills, NSW, Australia) instead of thiamine. One Centrum tablet was dissolved in 100 mL water, filter sterilised, and 10 ml added per litre of 3MN medium, post autoclaving. Peloton-rich tissues (collars) of *Chiloglottis* were washed several times with sterilised distilled water after which the tissue was macerated in sterilised distilled water to release pelotons which were plated onto agar plates containing antibiotics (FIM + tetracycline 25 mg/mL, and 3MN + A-Z + streptomycin 50 mg/mL). Germinating pelotons were transferred to either FIM or 3MN + A-Z media after 3–10 days. The media choice depended on which medium the pelotons germinated on. After 3–4 weeks all colonies were hyphal-tipped and subcultured to ensure colonies consist of a single genotype. Cultures were stored at 5 °C on FIM or 3MN + A-Z agar slants covered with mineral oil.

To investigate the presence of fungal species that could not be cultured *in vitro*, a portion of the *Chiloglottis* peloton-rich tissues (2 × *C*. aff. *valida*, 2 × *C*. *turfosa*, 2 × *C*. *gunnii*, *C*. *valida*, and *C*. *triceratops*) were stored at −80 °C for DNA extraction and direct sequencing (see below). One plant each of *C*. aff. *valida* and *C*. *turfosa* were growing in sphagnum hammocks. Rhizoids of *Sphagnum cristatum* or *S*. *novo-zelandicum* were also collected from mountain sphagnum hammocks (from Kosciuszko and Namadgi NP where *Chiloglottis* plants were collected) to assess *Tulasnella* presence and identity. As with *Chiloglottis* peloton-rich tissue, *Sphagnum* rhizoids were washed several times with sterilised distilled water to minimise the presence of saprophytic fungi on the surface. Voucher specimens of the fungi are stored in the culture collection of CCL laboratory at the Australian National University (ANU; see Supplementary Table S1).

#### Fungal DNA extraction and analysis

Small agar blocks cut from colony edges of isolates were gently homogenised in 2 ml screw-cap tubes containing sterilised distilled water and glass beads. The blocks were homogenised in a FP120 (Thermo Scientific) homogenizer for 15 sec at 4.0 m/s. Petri dishes containing either FIM or 3MN + A-Z broths were inoculated with the homogenised agar blocks and incubated at room temperature in the dark. Mycelium was harvested, stored at −4 °C and lyophilized prior to DNA extraction. DNA extraction of the lyophilized-mycelium, peloton-rich *Chiloglottis* tissues and *Sphagnum* rhizoids were performed using a Qiagen DNeasy Plant Mini Kit or DNeasy 96 Plant Kit according to the manufacturer’s instructions (Qiagen, Hilden, Germany).

#### Fungal ITS sequencing and phylogenetic analysis

In a comprehensive evaluation of eight nuclear and mitochondrial loci, Linde *et al*.^10^ showed that within *Tulasnella* a single locus, ITS (nuclear ribosomal internal transcribed spacer region), revealed congruent species delimitation and phylogenetic outcomes. Therefore, for the phylogenetic analysis in this study we only employed ITS. Details of ITS sequencing, phylogenetic analyses and diversity analyses can be found in the Supplementary Information.

### SSR genotyping and population genetic analyses

Ten Simple Sequence Repeat loci (SSR) specifically developed for *Tulasnella* isolates previously obtained from *Chiloglottis* orchids were amplified and genotyped as per Ruibal *et al*.^56^ for 253 isolates. Isolates where SSR amplification failed for some loci were re-amplified at least once to confirm null-alleles. Except where stated, GenAlEx version 6.501^50, 57^ was used to conduct the analyses as set out below.

#### Determination of Unique identity of Multi Locus Genotypes

The power of the SSR loci to uniquely identify individuals (given sample size) was assessed using theoretical predictions of the probability of identity for unrelated (*PI*) and related individuals (*PI*_*sibs*_). These analyses provide a theoretical prediction of the average probability that two individuals drawn from the same randomly mating population will by chance have the same multi-locus genotype. *PI* and *PI*_*sibs*_ estimates were calculated using the formula provided in Peakall and Smouse^57^.

#### Mating system

To assess the mating system for *Tulasnella*, Hardy Weinberg Equilibrium (HWE) tests were performed across loci within sites to assess departures from equilibrium. For this and the AMOVA analyses below, identical genotypes were removed from the dataset (hereinafter referred to as clone-corrected data). Heterozygote excess or deficit within each site was assessed with the global HWE test using Genepop version 4.2 (available online at URL: http://kimura.univ-montp2.fr/~rousset/Genepop.htm). *P*-values were estimated using the Markov chain method (10000 dememorisations, 100 batches, 5000 iterations per batch). To account for multiple comparisons, significance levels of both HWE and *F* were corrected for using Bonferroni corrections^58^.

#### Patterns of genetic structure associated with fungal identity, geographic origin, edaphic conditions and host identity

A hierarchical Analysis of Molecular Variance (AMOVA) was performed on the SSR data set to estimate the degree of genetic differentiation (*F*_*ST*_) and to partition genetic variation within and among *a priori* defined *Tulasnella* groups. These groups included: 1. Putative *Tulasnella* species predefined based on the outcome of the ITS phylogenetic analysis, 2. Sampling locations (populations) within species, 3. Within species grouped by edaphic conditions (soil versus sphagnum). These same three groupings were overlaid onto a minimum spanning network of the SSR MLGs. The network was constructed and visualized in R^59^ using the package igraph^60^ and the kamada.kawai layout option in the R package magrittr to display connections in 2D without overlap of edges in the graph. The network was constructed based on Bruvo’s distance^61^ which assumes a stepwise mutation model in the calculation of SSR genetic distances among individuals.

#### Fine-scale within site genetic structure

A multilocus autocorrelation method^62–64^ was used to test for local fine-scale spatial genetic structure. This method allows a test of the null hypothesis of a random distribution of genotypes in space, against the alternative hypothesis of a clustered (non-random) distribution of genotypes. Although the method is generic when analysing multi-allelic and multi-locus genetic data, the generated autocorrelation coefficient *r* is closely correlated with relatedness^65, 66^. This fine scale spatial genetic structure analysis was conducted at the within-site level at the site GST.

Mantel tests for isolation by distance were performed following Smouse *et al*.^67^, with pairwise geographic and individual genetic distances. Statistical significance of AMOVA and Mantel tests was assessed by 9999 random permutations.

## Electronic supplementary material

Supplementary information
