# Supplementary material for: Population structure of an orchid mycorrhizal fungus with genus-wide specificity
Source: Sci Rep. 2017 Jul 17;7:5613. doi: 10.1038/s41598-017-05855-3 (PMC5514033; doi:10.1038/s41598-017-05855-3)
Supplement: Supplementary file 1 — Supplementary information [file 41598_2017_5855_MOESM1_ESM.pdf]

Population structure of an orchid mycorrhizal fungus with genus-wide specificity

Ruibal, M. P., Triponez, Y., Smith, L. M., Peakall, R. and Linde, C. C.*

Evolution, Ecology and Genetics, Research School of Biology, The Australian National University, Canberra, ACT 2601, Australia.

Corresponding author. Email: celeste.linde@anu.edu.au

Supplementary material

Methods

Fungal ITS sequencing and phylogenetic analysis: For samples obtained from *Tulasnella* cultures, ITS sequences were amplified with the primers ITS1 and ITS4[1]. For DNA obtained from *Chiloglottis* and *Sphagnum* peloton-rich tissue, the *Tulasnella*-specific primer pair ITS1-ITS4-tul[1,2] and the fungal specific combination ITS1F and ITS4[1,3] were used. We followed methods described in Roche *et al.*[4] for the PCR reaction, thermal cycling, and purification of PCR and extension products. Products were sequenced bi-directionally with ABI PRISM BigDye Terminator v3.1 sequencing kit (Applied Biosystems, Foster City, California, USA) on an ABI-3131 automated sequencer. Sequences were edited using the program Sequencher version 4.7 (GeneCodes, Ann Arbor, Michigan, USA) to correct for base read ambiguities. Alignments were performed in Geneious version 8 (http://www.geneious.com)[5] using ClustalW followed by manual adjustments to optimise indel locations. Although a large number of isolates were obtained specifically for this study (see Table S1), we also included samples from Roche *et al.*[4], Linde *et al.*[6] and Linde *et al.*[7] in

the alignment. Representative sequences of *Tulasnella* outgroups were obtained from GenBank and included in the alignment. Estimates of variability were performed with Mega 5.05[8]. Phylogenies were estimated using Bayesian inference with Mr Bayes 3.1.2[9] and maximum likelihood (ML) analysis through the RAxML Blackbox[10]. Support for the nodes was assessed with Bayesian Posterior Probabilities (BPP) in MrBayes and for ML trees using 1000 pseudoreplicates of nonparametric bootstrapping. A GTR+G substitution model was used for all analyses as other models are nested within these. Trees were visualised using FigTree v1.4.2 (http://tree.bio.ed.ac.uk/software/figtree/) and mid-point rooted. Trees include identical sequences from different isolates; however the identical sequences were removed when nodes support was assessed. Previously obtained sequencing data for ITS from Roche *et al.*[4] and Ruibal *et al.*[11] were included in the analysis (Table S1). Sequence diversities and genetic divergence were calculated in Mega 5.05[8] among all isolates available (see Table S1 for complete list). For genetic divergence estimation we employed the *p*-distance as well as the Kimura 2-parameter (K2P) distances[12] (pairwise deletion).

Table S1. Fungal isolate collection details from which *Tulasnella* pelotons were isolated. Colony = plant colony code, *Chiloglottis* species = species of orchid fungus isolated from, Source = isolates collected by, MLG *T. prima* = number of genets identified with SSR's, No. of isolates *T. sphagneti* = number of isolates belonged to the species *T. sphagneti.* NSW = New South Wales, ACT = Australian Capital Territory, TAS = Tasmania,

Isolate	Site Name	Ecotype	Site Abbr.	*Chiloglottis species*	GPS	Collection Date	Region	No. of plants collected/No. of plants *Tulasnella* isolates obtained	Source	No. of *T. prima* MLGs	No. of *T. sphagneti* isolates
11142, 13051-56, 13166-70, 13107-11	Sawyer's Hut	Soil	SH	*C. turfosa*	S35.896 E148.539	19/01/2012 & 6/12/2013	Kosciuszko National Park, NSW	19 / 16	This study	6	0
12029-31, 11053, 11087, 13102-06, 13171	Sawyer's Hut	Sphagnum	SH	*C. turfosa*	S35.896 E148.539	19/01/2012 & 6/12/2013	Kosciuszko National Park, NSW	11 / 8	This study	4	3
11050-51, 11065, 11092, 13080-101	Alpine Creek	Soil	AC	*C.* aff. *valida* / *C. valida*	S35.9274 E148.592	19/01/2012 & 6/12/2013	Kosciuszko National Park, NSW	26 / 20	This study	10	0
13057-79	Alpine Creek	Sphagnum	AC	*C.* aff .*valida*/ *C. turfosa*	S35.927 E148.592	6/12/2013	Kosciuszko National Park, NSW	24 / 22	This study	5	12
11098, 11101, 11109, 13112-20, 13178-94, 13195-200	Wares Yard	Soil	WY	*C.* aff. *valida* / *C. turfosa*	S35.878 E148.618	6/12/2013	Kosciuszko National Park, NSW	36 / 24	This study	17	2
13172-77	Wares Yard	Sphagnum	WY	*C.* aff. *valida* / *C. turfosa*	S35.878 E148.618	6/12/2013	Kosciuszko National Park, NSW	7/ 7	This study	0	7
13159, 13162, 13165	Goldseekers Track	Soil	GST	*C. turfosa*	S35.892 E148.445	6/12/2013	Kosciuszko National Park, NSW	3 / 1	This study	14	0
11146, 11148, 12036, 13121-58, 13160, 13162-64	Goldseekers Track	Sphagnum	GST	*C. turfosa* *C.* aff. *valida*	S35.892 E148.445	19/01/2012 & 6/12/2013	Kosciuszko National Park, NSW	44 / 27	This study	6	3

12033-34	Tantangara Rd	Sphagnum	TTR	*C.* aff. *valida*	S35.8919 E148.623	19/01/2012	Kosciuszko National Park, NSW	2 / 2	This study	0	2
11052	Ghost Gully	Soil	GG	*C. turfosa*	S35.694 E148.592	31/12/2012	Kosciuszko National Park, NSW	4 / 3	This study	5	
12037, 12039-40, 12043	Corin Forest	Sphagnum	CF	*C. valida*	S35.519 E148.907	14/02/2012	Namadgi National Park, ACT	4 / 4	This study	1	
12044, 12047	Corin Forest	Soil	CF	*C. valida*	S35.519 E148.907	14/02/2012	Namadgi National Park, ACT	2 / 2	This study	1	
12024-25	Devils Kitchen	Soil	TAS10	*C. reflexa*	S43.0433 E147.950	7/01/2012	Tasman National Park, TAS	2 / 2	This study	1	
12022	Fortescue Rd	Soil	TAS9	*C. cornuta*	S43.108 E147.910	7/01/2012	Tasman National Park, TAS	1 / 1	This study	1	
12019-20	Pelham & Marked Tree Rd	Soil	TAS8	*C. gunnii*	S42.563 E146.959	6/01/2012	Hollow Tree, TAS	2 / 2	This study	1	
12011	Hobart	Soil	TAS6	*C. triceratops*	S42.899 E147.273	4/01/2012	Mt Wellington Park, TAS	1 / 1	This study	1	
12002	Mt Wellington 1	Soil	TAS1	*C. grammata*	S42.907 E147.243	4/01/2012	Mt Wellington Park, TAS	1 / 1	This study	1	
12004	Mt Wellington 3	Soil	TAS3	*C. grammata*	S42.902 E147.242	4/01/2012	Mt Wellington Park, TAS	1 / 1	This study	1	
12014	Pelverata Falls track	Soil	TAS7	*C. triceratops*	S43.0487 E147.129	4/01/2012	Snug Tiers Nature Recreation Area, TAS	2 / 1	This study	1	
11122, 11125-26	Pole Blue camp	Soil	PBC	*C. pluricallata / C. palachila*	S31.956 E151.425	8/12/2011	Barrington Tops National Park, NSW	3 / 3	This study	4	
11129-30, 11132	Pole Blue swamp	Sphagnum	PBS	*C.* sp. (bifaria)	S31.957 E151.427	8/12/2011	Barrington Tops National Park, NSW	3 / 3	This study	3	
	Wynnes Lookout		WL	*C. reflexa*		4/10/2006	Mt Wilson, Blue Mountains, NSW	3	Roche et al. 2010	3	
	Mt Irvine Rd		MI	*C. reflexa*		4/10/2006	Mt Wilson, Blue Mountains, NSW	1	Roche et al. 2010	1	
	Upper Kangaroo Valley		UKV	*C. formicifera*			Kangaroo Valley, NSW	3	Linde et al. 2014	1	
	Fitzroy Falls		FF	*C. seminuda*		31/03/2007	Meryla State Forest, NSW	3	Linde et al. 2014	2	
	Boyd River fire trail		BRFT	*C.* aff. *jeanesii*		29/10/2007	Kanangra Boyd National Park, NSW	5	Roche et al. 2010	5	
	Australian National Botanical		ANBG	*C. trapeziformis*		18/09/2008	Canberra, ACT	1	Roche et al. 2010	1	

	Gardens										
	Black Mountain		BM	*C. trapeziformis*		18/09/2008	Canberra, ACT	5	Roche et al. 2010	3	
	Bilpin Park		BP	*C. diphylla*		31/03/2007	Blue Mountains, NSW	4	Roche et al. 2010	3	
	Hanging Rock Rd		HR	*C. seminuda*		31/03/2007	Penrose Forest, Exeter, NSW	3	Roche et al. 2010	2	
	Lowden Rd.		LR	*C. valida*		4/10/2006	Tallaganda State Forest, NSW	5	Roche et al. 2010	4	
	Mt Werong		MW	*C. seminuda*		31/03/2007	Blue Mountains, NSW	4	Roche et al. 2010	4	
	Mt Werong fire trail		MtW	*C. trilabara*		31/03/2007	Blue Mountains, NSW	4	Roche et al. 2010	4	

Table S2. Outcomes of tests for Hardy-Weinberg equilibrium for *Tulasnella prima* from Kosciuzsko National Park sites.

Kosciuszko NP

Pop	Locus	DF	ChiSq	Prob	Signif
AC	Tul 2	6	14.02	0.029	*
AC	Tul 11	21	34.78	0.030	*
AC	Tul 12	3	6.12	0.106	ns
AC	Tul13	3	13.10	0.004	**
AC	Tul 16	15	38.30	0.001	***
AC	Tul 17	1	0.19	0.667	ns
AC	Tul 23	10	42.56	0.000	***
AC	Tul 24	Monomorphic			
AC	Tul 65	Monomorphic			
AC	Tul TGC6	6	16.69	0.010	*
GG	Tul 2	1	0.06	0.804	ns
GG	Tul 11	6	5.56	0.475	ns
GG	Tul 12	1	2.22	0.136	ns
GG	Tul13	1	5.00	0.025	*
GG	Tul 16	1	4.00	0.046	*
GG	Tul 17	1	0.06	0.804	ns
GG	Tul 23	6	5.00	0.544	ns
GG	Tul 24	Monomorphic			
GG	Tul 65	1	0.06	0.804	ns
GG	Tul TGC6	3	5.10	0.164	ns
GST	Tul 2	6	40.00	0.000	***
GST	Tul 11	6	52.31	0.000	***
GST	Tul 12	6	30.46	0.000	***
GST	Tul13	21	73.50	0.000	***
GST	Tul 16	36	66.86	0.001	**
GST	Tul 17	6	20.14	0.003	**
GST	Tul 23	10	40.44	0.000	***
GST	Tul 24	1	15.96	0.000	***
GST	Tul 65	1	20.00	0.000	***
GST	Tul TGC6	15	59.77	0.000	***
SH	Tul 2	10	5.62	0.846	ns
SH	Tul 11	15	12.07	0.674	ns
SH	Tul 12	21	23.67	0.309	ns
SH	Tul13	3	1.11	0.774	ns
SH	Tul 16	15	23.03	0.084	ns
SH	Tul 17	3	12.49	0.006	**
SH	Tul 23	3	6.52	0.089	ns
SH	Tul 24	Monomorphic			
SH	Tul 65	1	0.03	0.868	ns
SH	Tul TGC6	3	1.93	0.588	ns
WY	Tul 2	3	18.26	0.000	***
WY	Tul 11	6	15.85	0.015	*
WY	Tul 12	6	18.99	0.004	**
WY	Tul13	6	15.15	0.019	*

WY	Tul 16	21	39.11	0.010	**
WY	Tul 17	3	17.12	0.001	***
WY	Tul 23	6	35.40	0.000	***
WY	Tul 24	1	17.00	0.000	***
WY	Tul 65	1	0.07	0.790	ns
WY	Tul TGC6	6	15.52	0.017	*

Table S3. Outcomes of tests for Hardy-Weinberg equilibrium for *Tulasnella sphagneti* from Kosciuszko National Park sites.

Site	Locus	DF	ChiSq	Prob	Signif
AC	Tul 2	Monomorphic			
AC	Tul 11	6	14.000	0.030	*
AC	Tul 12	1	7.000	0.008	**
AC	Tul 16	Monomorphic			
GST	Tul 2	Monomorphic			
GST	Tul 11	1	3.000	0.083	ns
GST	Tul 12	1	3.000	0.083	ns
GST	Tul 16	Monomorphic			
SH	Tul 2	Monomorphic			
SH	Tul 11	1	0.918	0.338	ns
SH	Tul 12	3	5.000	0.172	ns
SH	Tul 16	1	0.062	0.804	ns
WY	Tul 2	Monomorphic			
WY	Tul 11	3	3.645	0.302	ns
WY	Tul 12	1	9.000	0.003	**
WY	Tul 16	Monomorphic			

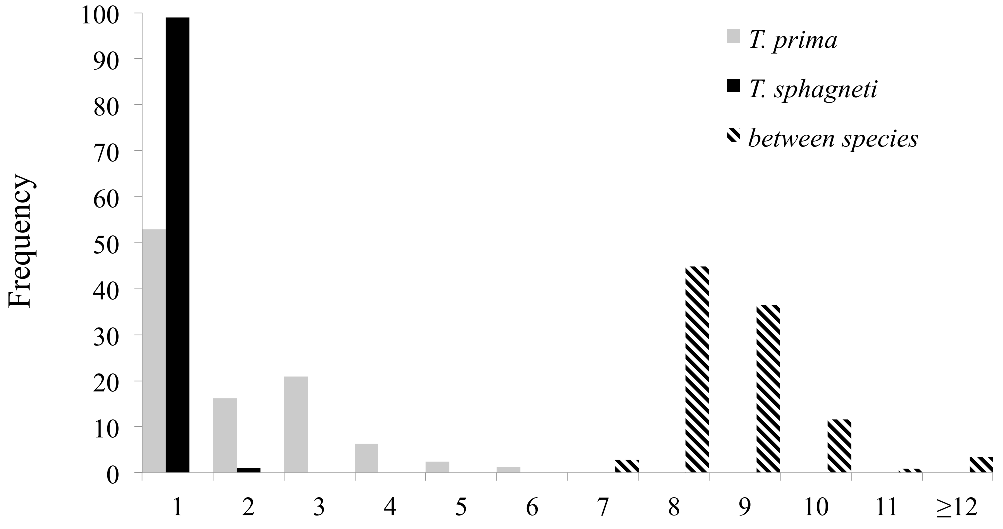

Figure S1. Frequency distribution of percentage of pairwise diversity (KP2) within species and between species of *Tulasnella* fungi.

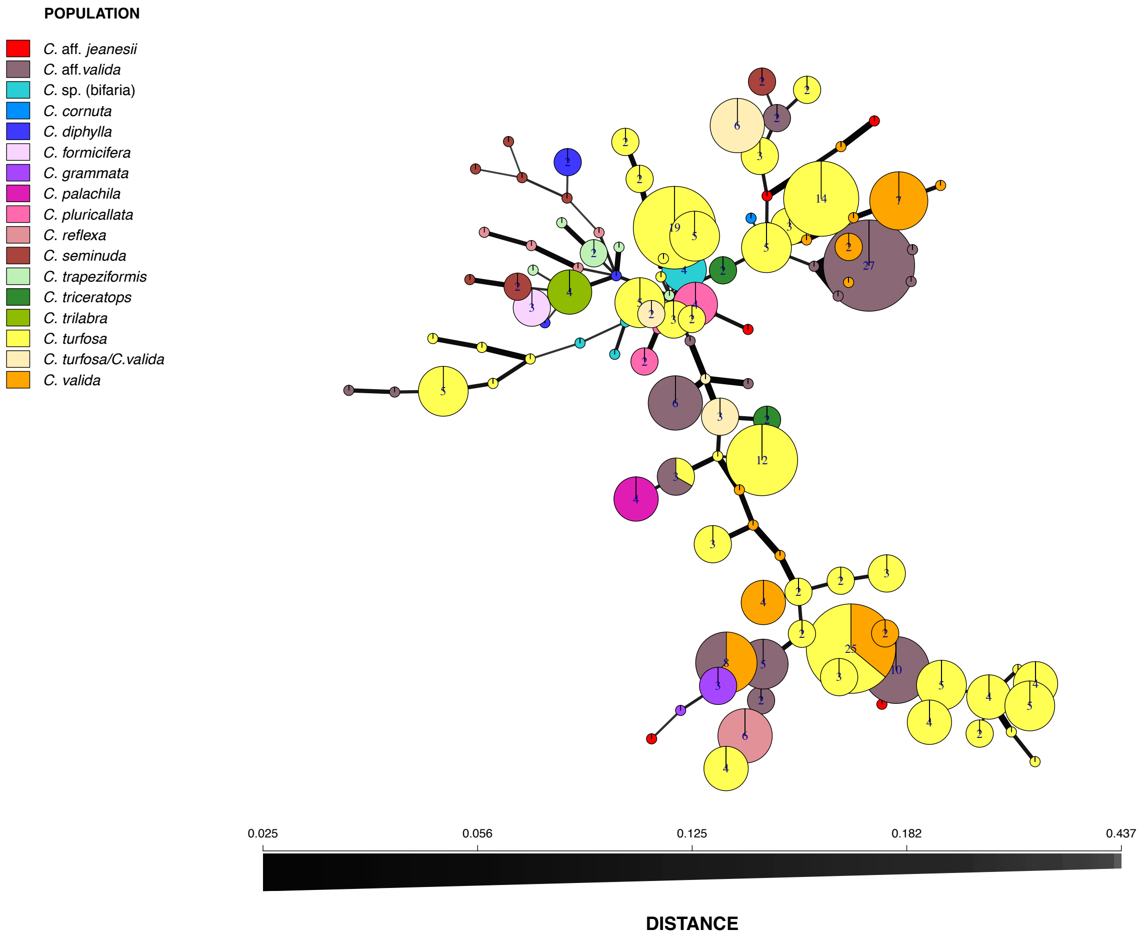

Figure S2. Minimum spanning network showing the relationship between the *Chiloglottis* orchid species and individual SSR multilocus genotypes (MLG) for *Tulasnella prima* observed in isolates collected from NSW, ACT and Tasmania. Each node represents a different MLG. Node sizes and colours correspond to the number of isolates and membership, respectively. The number of isolates per MLG greater than one is provided. Edge thickness and colour are proportional to absolute genetic distances.

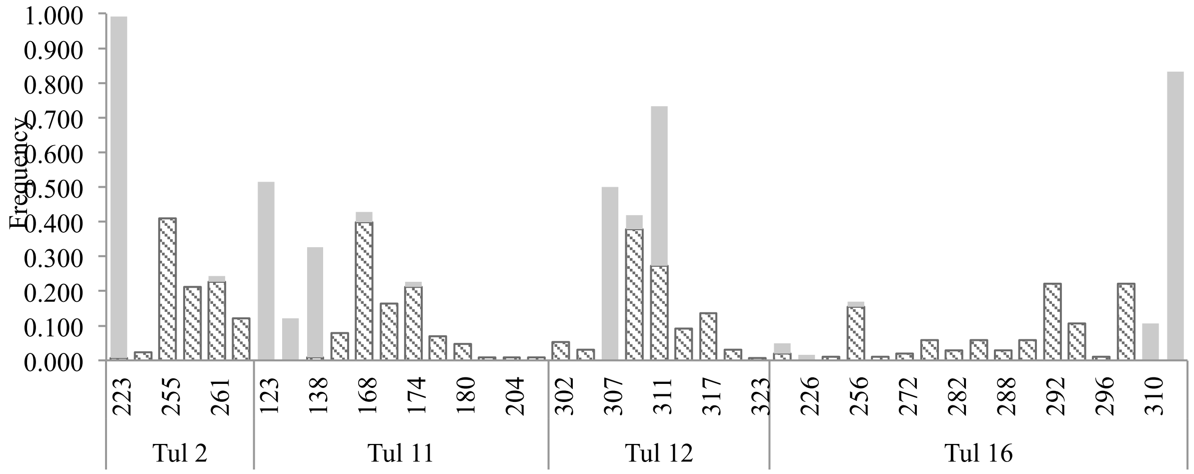

Figure S3. Comparison of allelic frequency recorded at four SSR loci shared between *Tulasnella prima* (striped bars) and *Tulasnella sphagneti* (solid bars) for Kosciuszko NP isolates.

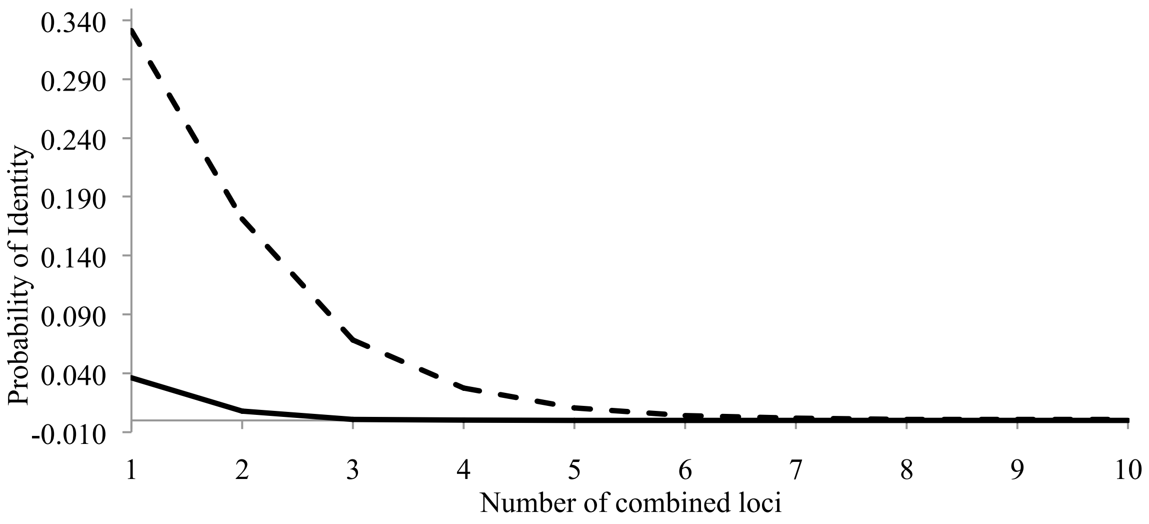

Figure S4. The theoretical predictions of the probability of identity for increasing combinations of loci for unrelated (PI solid line) and related (PI_{sibs} broken line) isolates collected from Kosciuszko National Park. Loci were combined from the most variable to the least variable (Tul 16, Tul 23, TulTGC, Tul 13, Tul 11, Tul 12, Tul 2, Tul 17, Tul 24, Tul 65). The criterion for variability was determined by the number of alleles and then expected heterozygosity for each locus across all unique multi-locus genotypes.

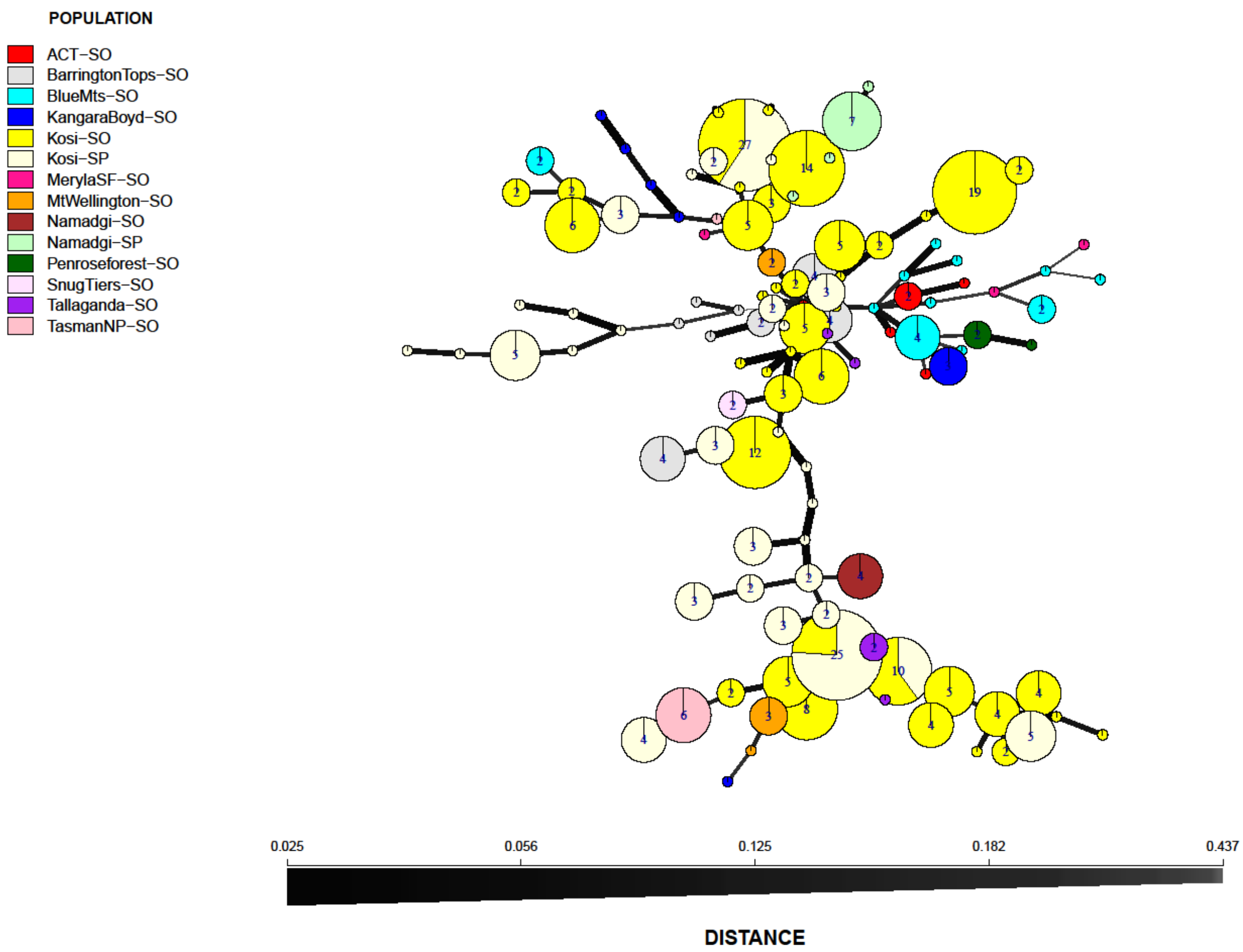

Figure S5. Minimum spanning network showing the relationship between individual SSR multilocus genotypes (MLG) for *Tulasnella prima* observed in isolates collected from NSW, ACT and Tasmania. Each node represents a different MLG. Node sizes and colours correspond to the number of isolates and membership, respectively. The number of isolates per MLG greater than one is provided. Edge thickness and colour are proportional to absolute genetic distances.

References

1 White, T. J., Bruns, T., Lee, S. & Taylor, J. W. in *PCR protocols: a guide to methods and applications* (eds M. A. Innis, D. H. Gelfand, J. J. Sninsky, & T. J. White) 315-322 (Academic Press, 1990).

2 Taylor, D. L. *The evolution of myco-heterotrophy and specificity in some North Amercian orchids*. (University of California at Berkeley, Berkeley, CA, 1997).

3 Gardes, M. & Bruns, T. D. ITS primers with enhanced specificity for basidiomycetes – application to the identification of mycorrhizae and rusts. *Molecular Ecology* **2**, 113-118 (1993).

4 Roche, S. *et al.* A narrow group of monophyletic *Tulasnella* (Tulasnellaceae) symbiont lineages are associated with multiple species of *Chiloglottis* (Orchidaceae): Implications for orchid diversity. *American Journal of Botany* **97**, 1313-1327 (2010).

5 Kearse, M. *et al.* Geneious Basic: an integrated and extendable desktop software platform for the organization and analysis of sequence data. *Bioinformatics* **28**, 1647-1649 (2012).

6 Linde, C. C., Phillips, R. D., Crisp, M. D. & Peakall, R. Congruent species delineation of *Tulasnella* using multiple loci and methods. *New Phytologist* **201**, 6-12 (2014).

7 Linde, C. C. *et al.* New species fo *Tulasnella* associated with terrestrial orchids in Australia. *IMA Fungus* **8**, 27-47 (2017).

8 Tamura, K. *et al.* MEGA5: Molecular Evolutionary Genetics Analysis using Maximum Likelihood, Evolutionary Distance, and Maximum Parsimony Methods. *Molecular Biology and Evolution* **28**, 2731-2739 (2011).

9 Ronquist, F. & Huelsenbeck, J. P. MrBayes 3: Bayesian phylogenetic inference under mixed models. *Bioinformatics* **19**, 1572-1574 (2003).

10 Stamatakis, A., Hoover, P. & Rougemont, J. A Rapid Bootstrap Algorithm for the RAxML Web Servers. *Systematic Biology* **57**, 758-771 (2008).

11 Ruibal, M. P., Peakall, R., Smith, L. M. & Linde, C. C. Phylogenetic and microsatellite markers for *Tulasnella* (Tulasnellaceae) mycorrhizal fungi associated with Australian orchids. *Applications in Plant Sciences* **1**, 1200394 (2013).

12 Kimura, K. A simple method fo estimating evolutionary rates of base substitutions through comparative studies of nucleotide sequences. *Journal of Molecular Evolution* **16**, 111-120 (1980).
